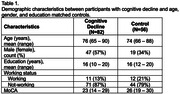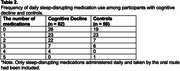# Quantitative effects of sleep‐disrupting medications on real‐world sleep metrics in older adults at risk for neurodegeneration

**DOI:** 10.1002/alz.089806

**Published:** 2025-01-03

**Authors:** Jun Ha Chang, Anthony J. Scalzo, Christopher L. Shaffer, Matthew Rizzo

**Affiliations:** ^1^ University of Nebraska Medical Center, Omaha, NE USA

## Abstract

**Background:**

This pilot study addresses unmet needs for empirical evidence on real‐world data (RWD) on sleep to inform pharmacological management in older adults at‐risk for neurodegenerative conditions. Polypharmacy is prevalent among older adults, with potential adverse effects on physiological functions, including sleep. Sleep disturbances are prevalent in aging, may signal onset of Alzheimer’s disease (AD), potentially contributing to the underlying pathology.

**Methods:**

82 participants identified with cognitive decline (9 mild AD, 73 Mild Cognitive Impairment) (mean age = 76yrs; 47 male) and 56 age‐ and education‐matched controls (mean age = 75yrs; 19 male) (Table 1) self‐reported their prescribed medications. Only medications known to disrupt sleep (e.g., Cholinesterase inhibitors, ACE inhibitors) and administered orally daily were included in the analysis. Cognitive abilities were measured in all 138 participants at study induction using a battery of standard neuropsychological tests. Four weeks of nightly sleep data were collected using wrist‐worn actigraphy to estimate sleep duration and quality, including sleep efficiency [SE], wakefulness after sleep onset [WASO], and awakening count [AC]). Multivariate linear regressions examined the effects of the number of sleep‐disrupting medications and cognitive status on sleep variables, adjusting for age, gender, and education.

**Results:**

45 participants did not take sleep‐disrupting medications, 46 took one, and 47 took two or more (Table 2). Greater numbers of the medications were linked to poorer SE (b = ‐0.26, p = 0.01), increased WASO (b = 0.27, p = 0.01), and greater variability in sleep duration (b = 0.23, p = 0.03), SE (b = 0.26, p = 0.02), WASO (b = 0.28, p = 0.009), and AC (b = 0.26, p = 0.01). Controls showed less variability in SE (b = ‐0.43, p = 0.01) and WASO (b = ‐0.37, p = 0.03) than those with cognitive decline.

**Conclusion:**

Our results, using RWD of sleep and medications, underscore that polypharmacy in older adults is associated with disrupted sleep patterns, with a greater number of sleep‐disrupting medications exacerbating poor sleep outcomes. These findings highlight needs for careful medication management to preserve sleep and suggest that targeted pharmacological strategies can mitigate medication‐related sleep disturbances, thereby enhancing brain health, behavior, and overall health.